# Spinal Hyper-Excitability and Altered Muscle Structure Contribute to Muscle Hypertonia in Newborns After Antenatal Hypoxia-Ischemia in a Rabbit Cerebral Palsy Model

**DOI:** 10.3389/fneur.2018.01183

**Published:** 2019-01-17

**Authors:** Sylvia Synowiec, Jing Lu, Lei Yu, Ivan Goussakov, Richard Lieber, Alexander Drobyshevsky

**Affiliations:** ^1^Department of Pediatrics, NorthShore University HealthSystem Research Institute, Evanston, IL, United States; ^2^Department of Pediatrics, University of Chicago, Chicago, IL, United States; ^3^Department of Physical Medicine and Rehabilitation, Northwestern University and the Shirley Ryan Ability Lab, Chicago, IL, United States

**Keywords:** cerebral palsy, spinal excitability, muscle mechanical property, motoneurons, perinatal brain injury

## Abstract

Rabbit kits after global antenatal hypoxic-ischemic injury exhibit motor deficits similar to humans with cerebral palsy. We tested several mechanisms previously implicated in spinal hyper-excitability after perinatal brain injury that may explain muscle hypertonia in newborns. Stiffness of hind limb muscles during passive stretch, electromyogram, and spinal excitability by Hoffman reflex, were assessed in rabbit kits with muscle hypertonia after global hypoxic-ischemic brain injury and naïve controls. Affected muscle architecture, motoneuron morphology, primary afferents density, gliosis, and KCC2 expression transporter in the spinal cord were also examined. Decrease knee stiffness after anesthetic administration was larger, but residual stiffness was higher in hypertonic kits compared to controls. Hypertonic kits exhibited muscle shortening and atrophy, in both agonists and antagonists. Sarcomere length was longer in tibialis anterior in hypertonic kits than in controls. Hypertonic kits had decreased rate dependent depression and increased H_max_/M_max_ in H-reflex. Motor neuron soma sizes, primary afferent density were not different between controls and hypertonic kits. Length of dendritic tree and ramification index were lower in hypertonic group. Gene expression of KCC2 was lower in hypertonic kits, but protein content was not different between the groups. In conclusion, while we found evidence of decreased supraspinal inhibitory control and increased excitability by H-reflex that may contribute to neuronal component in hypertonia, increased joint resistance to stretch was explained predominantly by changes in passive properties of muscles and joints. We did not find structural evidence of increased sensory afferent input or morphological changes in motoneurons that might explain increased excitability. Gliosis, observed in spinal gray matter, may contribute to muscle hypertonia.

## Introduction

Cerebral palsy (CP) is a syndrome of motor impairment that results from an injury occurring in the developing brain with prevalence of 2.0–3.5 per 1,000 live births (1). Cerebral palsy constitutes an “umbrella” term for disorders with variable manifestation of motor deficits (2, 3) due to inhomogeneous injury sites and variable timing of injury occurring in early perinatal period and affecting later development (4–6).

Perinatal brain injury due to hypoxia, ischemia, and/or inflammation (7–9) is commonly described as the etiology of CP (1). Mechanisms contributing to motor dysfunction in CP remain poorly understood and likely involve changes in descending motor control as well as altered spinal circuitry and intrinsic properties of spinal motoneurons due to abnormal cord development (3, 10).

Muscle hypertonia is common in CP and is characterized by increased resistance to joint movement (11). CP motor phenotype is commonly attributed to upper motor neuron syndrome, originating from injury to sensorimotor cortex or descending white matter projections (11–13). Positive features of upper motor neuron syndrome are often considered as the origin of spinal hyperreflexia, muscle spasticity and co-contractions in CP (3, 11). Spasticity and enhanced motoneuron activity are important causes of hypertonia, but increased tone may also be attributable to altered passive tissue properties of muscles and joints or the inability to relax the muscle prior to initiation of movement (3, 14).

The most commonly described mechanisms thought to contribute to exaggerated motor cell activity in spinal cord injury and CP are reduced supraspinal inhibitory synaptic input (15), increased excitatory synaptic input due to decreased retraction of primary afferents occurring in normal development and regulated by descending motor projections (10, 16–18), loss of segmental inhibitory interneurons (19), loss of recurrent inhibition mediated by Renshaw cells (20). Animal studies suggest that that increased intrinsic motoneuron excitability after spinal cord injury plays a role in CP and can result from changes in membrane metabotropic receptors properties (21) and ion transporters, such as potassium-chloride transporter [KCC2; (22)], as well as the morphology of motoneurons soma and dendritic tree (23, 24).

While all or some of the above mechanisms may play a role in human CP, the lack of animal models with overt motor deficits after developmental injury impedes testing them at the cellular and molecular level. The rabbit global antenatal hypoxic-ischemic (H-I) injury model captures several salient features of human CP, including pronounced muscle hypertonia, locomotor, postural and sensory deficits in newborn rabbit kits (25, 26). White and gray matter brain injury in this model, resulting in a decrease of descending motor projections, may represent a mechanism that leads to the development of motor deficits (27, 28). In the current study we examined several other mechanisms that may explain muscle hypertonia in the rabbit model as well as the muscle structural changes resulting from the injury.

Since most newborn rabbit kits after antenatal global H-I exhibit characteristic posture at rest consisting of exaggerated flexion in distal forelimbs and extension in distal hind limbs joints, both of which are characteristic of patients with spastic CP (12), we first determined whether postural deficits were caused by active neuronal component, i.e., by increased persistent muscle activity and imbalance between flexor and extensors muscle groups. Increased persistent muscle activity at rest could be attributed to increased tonic discharge from motoneurons without explicit stimulation. Alternatively, postural deficits could be attributed to structural changes and alteration in biomechanical properties of muscles and joints (29).

Second, we determined whether increased joint resistance in newborn rabbits after global antenatal H-I injury was associated with spinal hyper-excitability due to decreased supraspinal presynaptic inhibition, implicated as a mechanism underlying spasticity in cerebral palsy (15, 30). To address this mechanism, we measured the Hoffman (H-) reflex, an electric analog of stretch reflex, during normal development and after global antenatal H-I injury. Abnormal increase of H-wave and decrease of rate dependent depression (RDD) of H-reflex is attributed to reduced supra-segmental inhibition after brain and spinal cord injury (31), with abnormalities also observed in children with CP (32, 33).

Finally, we examined the morphological and biochemical properties of motoneurons and glia (34) that may indicate increased excitability and functional activity, such as size of soma and dendritic tree (23, 24) and expression of KCC2 transporter (35).

## Methods

The study was approved by the Institutional Animal Care and Use Committees of NorthShore University HealthSystem and was conducted in accordance with the United States Public Health Service's Policy on Humane Care and Use of Laboratory Animals.

### Animal Model

The surgical procedure has been previously described (25). New Zealand White pregnant rabbits (Charles Rivers, Canada) at embryonic day (E) 25 (78% term) underwent sustained uterine ischemia, modeling acute placental insufficiency at a premature gestation. Briefly, dams were anesthetized with intravenous fentanyl (75 μg/kg/hr) and droperidol (3.75 mg/kg/hr), followed by spinal anesthesia using 0.75% bupivacaine. A balloon catheter was introduced into the left femoral artery and advanced though the descending aorta to above the uterine and below the renal arteries. The balloon was inflated for 40 min causing uterine ischemia and subsequent global fetal H-I. At the end of H-I, the balloon was deflated, resulting in uterine reperfusion and reperfusion-reoxygenation to fetuses. The catheter was removed, the femoral artery reconstructed, and the dam returned to her cage. Sham control dams underwent the same surgical procedure, including balloon catheter insertion, but without the balloon inflation and did not receive uterine ischemia. The dams were allowed to deliver in a nest box at term (31.5 days).

At postnatal day 1 (P1), the kits underwent neurobehavioral testing to determine the extent of motor and sensory deficits, including muscle tone measurements (25). Tone was assessed by passive flexion and extension of the forelimbs and hind limbs and scored according to the modified Ashworth scale relative to age matched controls in corresponding limbs: 1—decreased tone/flaccidity, 2—normal tone, 3—moderate increase in tone but limb easily flexed, 4—limb rigid in flexion or extension. Hypertonic kits (muscle tone score >2 at least in one limb) after H-I and sham control kits were selected for examinations. Kits after H-I with normal muscle tone or flaccidity at P1 were not included in this study. One or two kits per litter were included in analysis.

Immediately after neurobehavioral testing the kits were euthanized and spinal cord tissue were processed for analysis, as described below.

### Muscle Tone Assessment

To evaluate contribution of active (neuronal) and passive (due to structural changes in muscle and tendons) components in muscle tone, passive joint resistance to stretch was assessed in ankles and knees in a subset of P1 kittens using a custom laboratory device as described in detail previously (36). Number of kits used for the muscle tone measurement was for ankle *n* = 13, 9, and for knee *n* = 16, 7, control and hypoxia, correspondingly. Joints were attached to a rotating jig and underwent repetitive flexion and extension cycles at 40 degrees maximum amplitude and 0.8 Hz frequency. Rotation angle and applied force were acquired using sensors. The first 15 s of stretch was not recorded to allow animal to acclimate to the apparatus and minimize voluntary muscle resistance. Joint stiffness, as a measure of muscle tone, was derived from linear regression of the displacement–torque curves. After the initial measurement, the kits were administered i.m. Ketamine 35 mg/kg and Xylazine 5 mg/kg, an anesthesia regimen that provides surgical level of anesthesia and, after 25 min, the second muscle tone measurement was conducted.

### Muscle Architecture Determination

After sacrifice at P1, limbs of controls and hypertonic kits (*n* = 5, 4) were skinned, and fixed for at least 5 days in 10% buffered formalin in natural relaxed position, preserving posture/contractures position, if present. Ankle dorsiflexors—the tibialis anterior (TA) and extensor digitorum longus (EDL), and ankle plantar flexors—the medial gastrocnemius (GM) and soleus were carefully isolated and removed, blotted dry, and weighed. Muscle length was measured with a dial caliper as the distance from the origin of the most proximal muscle fibers to the insertion of the most distal fibers. Bundles consisting of 5–15 fibers were teased from the proximal, middle, and distal region of each muscle. Fiber bundle length (FL) was measured in each region. Physiological cross-sectional area (PCSA) was calculated according to (37) using muscle mass, fiber length and pinnation angle. Fiber bundles were frozen and sectioned along the fiber direction on a cryostat. Twenty micrometers of sections were mounted on slides, stained for H&E and sarcomere length was measured under 40x magnification. A subset of hind limbs was cross—sectioned on cryostat 20 μm thick and processed using trichrome stain (Masson) kit (Sigma-Aldrich, USA) according to manufacture protocol for connective tissue assessment. In this preparation cytoplasm and muscle fibers stain red whereas connective tissue displays blue coloration.

### H-reflex Testing and EMG Recording

Hoffman reflex was tested in P1, P5, P11, P18, P30, and adult naïve control rabbits (*n* = 8, 4, 5, 3, 2, 2) and in P1 and P11 (*n* = 5, 5) rabbit kits with muscle hypertonia after antenatal H-I. Animals were lightly anesthetized with Ketamine (20 mg/kg). To minimize effect of surgical procedure, we employed a recording method introduced by Ho and Waite (38). A concentric needle bipolar electrode was inserted transcutaneously for stimulation in the rostral /distal aspect of brachial plexus in the vicinity of radial/common trunk of medial/ulnar nerves in forelimbs (39) and in the vicinity of sciatic nerve in hind limbs. For recording, 100 micron diameter teflon coated wire electrodes with 1 mm exposed barbed tip were placed in m. flexor carpi radialis in forelimbs and m. gastrocnemius in hind limbs using a hypodermal needle. Nerve stimulation was delivered and electromyographic (EMG) responses were recorded using physiological monitor (MP-150, Biopac, CA). Stimulation consisted of bipolar square pulse 0.2 ms duration delivered through a stimulus isolator. EMG response was amplified x1,000 and band pass filtered 1 Hz−5 kHz. Maximum amplitude ratio of H-wave and M-wave (H_max_/M_max_) was measured by ramping stimulation pulse amplitude up from 0.05 to 1 V in 0.05 V increments with 30 s between the stimulation pulses. This sequence was repeated 3 times with 3 min rest between repetitions. Rate dependent depression (RDD) of the H-reflex was measured as a ratio of H-wave magnitudes in control and test stimuli pairs with amplitudes at the level to elicit maximum magnitude of H-wave, determined in the previous ramp experiment. Control and test stimuli pairs were presented every 10 s with 5, 2, 1, 0.5, 0.14, 0.03 s inter-stimulus intervals. The sequence was repeated 3 times with 3 min rest between repetitions. In some animals H-reflex testing was repeated at different ages. At the end of selected terminal experiments, H-reflex was measured on exposed nerve preparation with subsequent nerve cutting to confirm the origin of H-wave. Nerve conduction velocity was measured by the dividing distance between stimulating and recording electrodes by the time from the stimulation artifact and peak of the M-wave.

EMG in resting flexor and extensor muscles in hind limbs was recorded prior to H-reflex measurement with the setting as described above. In a subset of experiments, 2 sets of wire EMG electrodes were inserted in m. tibialis anterior and m. gastrocnemius. Recording was conducted in a baseline condition of resting muscles without limb motion before and 10 min after sedation by i.m. injection of Ketamine (35 mg/kg) and Xylazine (5 mg/kg) mixture. Root mean square of the EMG signal was derived with time window for signal averaging 0.03 s.

### Sensory Afferent and Motor Neuron Labeling

Retrograde neuronal tracer cholera toxin B (CTB) conjugated with Alexa Fluor 488 and 555 fluorophores (Invitrogen, cat. # C34775, C34776) was injected 3 μL 0.02 mg/mL in flexor and extensor muscle groups in wrists and ankles at P1 using Hamilton syringe. Injection was done in 3 locations for a muscle group to extend labeling coverage. Seven control and 11 hypertonic kits were used for tracing experiments. Two days after the tracer injection, animals were transcardially perfused with 4% paraformaldehyde solution, spinal cords extracted, postfixed, cryoprotected in 30% sucrose and frozen on dry ice. Serial sections 40 μm thick, 400 μm apart, of the cervical and lumbar spinal cord expansions were cut on a cryostat and mounted onto poly-lysine-coated slides (Sigma Aldrich, St Louis, MO, USA). Sections were visualized under a microscope (Leica Microsystems, Wetzler, Germany) attached to a motorized stage (Ludl Electronic Products, Hawthorne, NY). Photographs of the labeled motor neurons, at least 20 neurons per region, were taken under 40x magnification at focal depth where motoneuron cross-section area was the largest. Neuron soma boundaries were manually traced using ImageJ software and the area of the labeled motor neurons soma was calculated and reported as a proxy for motor neurons soma size. In addition, to reveal somatotopic organization and spinal localization, CTB labeling was done on a separate cohort of control kits (*n* = 6) by injection of the CTB tracers of different colors in proximal (shoulder, hip) and distal (elbow, ankle) muscle groups, flexors and extensor separately. Rostro-caudal and mediolateral distribution of labeled motoneurons was recorded on systematic serial sections relative to segmental nerves and spinal section anatomical landmarks.

To estimate the total length of labeled primary afferent fibers in the spinal cord we used spherical probe in Stereo-Investigator software (MBF Bioscience, Williston, VT, USA). CTB labeled primary afferents, forming distinctive patches in dorsal horn and in the intermediate CTB-labeled fibers were counted in cervical and lumbar expansions where labeling was present, for each kit on five 40-μm coronal sections that were 200 μm apart. Sampling grid of 100 × 100 μm^2^ was used. For all the samples, the virtual sphere was maintained at a radius of 10 μm and guard zones were 2μm. Contours were drawn on 10x magnification around the labeled areas in the dorsal horn and intermediate zone separately and counting was done on 40x. The length of the CTB-labeled fibers inside the volume of 1 μm^3^ in the spinal cord was calculated by dividing the total estimated length by the total measured volume.

### Golgi Stain and Morphological Analysis of Motor Neurons

Golgi–Cox staining was carried out on 150-μm-thick fresh sections of cervical and lumbar cord expansions, using the FD Rapid Golgi Stain Kit (FD NeuroTechnologies, USA) according to the manufacturer's protocol. Forty well-individualized (8 cells from 5 animals per group), well-impregnated neurons of rhomboid shape and some size >20 μm, with no fragmentation of staining, and most of the dendritic field appeared to be confined to one section, were measured for each one of the areas of motor neurons locations projecting to distal muscle groups [as determined by CTB tracing described above and according to the previous anatomical studies in rabbits (40)]. Stack of sections, covering dendritic tree throughout the slice, were captured (20x objective) at 1-μm intervals and maximum intensity projection image was obtained from the image stack using MetaMorph software (Molecular Devices, USA). Dendrites of each selected neuron were manually traced and the extent and distribution of dendritic branching of these neurons were evaluated by Sholl analysis (method of concentric circles) using ImageJ software (http://imagej.net/Sholl). Total length of dendrites, mean number of intersections on the distance 200 micron from cell body and ramification index (a measure of ramification, the ratio between maximum number of intersections and the number of primary branches) were calculated.

### Immunostaining and Stereological Estimation of Gliosis in Spinal Cord

Newborns P1 control rabbit kits (*n* = 6) and with hypertonia (*n* = 6) after H-I were anesthetized with Ketamine -Xylazine mixture and transcardially perfused with 0.1 M PBS, followed by 4% paraformaldehyde in 0.1 M PBS. Spinal cords were removed, post-fixed in the same fixative for at least 24 h, cryoprotected with 30% sucrose and frozen. Serial sections 40 μm thick were cut at 400 μm intervals on a cryostat from cervical (C4–C8) and lumbar expansions (L4–L7) and mounted onto poly-lysine-coated slides (Sigma Aldrich, USA) and air dried. Antigen retrieval was performed by heating samples in 10 mM sodium citrate pH 6.0 at 95°C for 15 min. The sections were blocked with 5% goat serum followed by incubation with the primary antibodies overnight at 4°C and biotinylated secondary antibody for 1h at room temperature, followed by avidin-biotin complex (Vectastain ABC kit, Vector Laboratories, Burlingame, CA, USA) for 1h. Color was developed using 3,3′-diaminobenzidine (Sigma Aldrich, MO, USA).

For immunostaining of astroglia, primary antibodies were chicken polyclonal anti-GFAP antibody (Abcam ab 4674, 1:200). The secondary antibodies were goat anti-chicken IgG (BA-9010, 1:200; Vector Laboratories). For immunostaining of microglia, primary antibodies were goat anti-Iba-1 (1:500, Abcam ab5076). The secondary antibodies were donkey anti-goat (1:200; Abcam ab6884).

Astroglia in spinal cord on GFAP staining presents as mostly glial processes with few cell bodies. Spherical ball probes were used to estimate the total length of GFAP-positive fibers in Stereo-Investigator. White and gray matter contours were outlined on the sections under 5x objective as reference space and fibers were counted under a 40x objective. Sampling grid of 250 × 250 μm^2^ was used. For all the samples, the virtual sphere was maintained at a radius of 10μm and guard zones were 2μm. The length of the GFAP-positive fibers inside the volume of 1 μm^3^ in the spinal cord was calculated by dividing the total estimated length by the total measured volume.

Numbers of Iba-1-positive cells were quantified in cervical and lumbar spinal cord expansions using optical fractionator probe in StereoInvestigator. White and gray matter contours were outlined on the sections under 5x objective as reference space. The cells were counted under a 10x objective with a counting grid 150 × 150 μm^2^, sampling grid 400 × 400 μm^2^. The Gundersen coefficient of error of the stereological estimation for each animal ranged from 0.05 to 0.12. The density of the Iba-1-positive cells was calculated as the total estimated number of cells divided by the measured volume.

### Real Time PCR

Kits from control and hypertonia groups (*n* = 5 from 2 litters, 5 from 4 litters, respectively) were sacrificed at P1. The cervical and lumbar cords were quickly harvested and stored in RNAlater solution (Life technologies, Grand Island, NY, USA). Rabbit KCC2 transporter PCR target sequence was based on the common part of alignment of human (NM_000868), mice (NM_008312) and rat (NM_012765) KCC2 transporter sequences, forward primer: 5′-GCT TCT ACT TGG GCA CTA CC-3′, and reverse primer, 5′-CCA TAG CTG GGA AGA GGT AAG−3′, probe ACA TCC TGG GCA CCA TCG AAA TCC. The sequence was then submitted to Applied Biosystems website to order custom made Taqman primer and probe set. RNA was reverse transcribed to cDNA using high capacity RT cDNA kit (ABI). Real time PCRs were carried in ABI StepOne machine with default program. 18s rRNA was used as an internal control. Relative quantification (RQ) values are presented.

### Western Blotting

Cord samples were obtained from control and hypertonia kits (*n* = 6, 5) at P1. To build developmental profile, 5 control kits were used to obtain cord samples at each age at E26, P1,P5,P11, P18, P25, and adults. Content of KCC2 were determined by Western blot using mouse monoclonal antibody (MAB8369, R&D, USA, dilution 1:500). Tissue samples were homogenized in ice-cold 1x RIPA lysis buffer. Equal amount of protein lysate was subjected to SDS-PAGE electrophoresis using Bio-Rad Criterion™ XT 4012% Bis-Tris precast gels (Bio-Rad Laboratories, Inc., Hercules, CA) and transferred to PVDF membranes using a semi-dry transfer system (Bio-Rad). The membranes were blocked with 5% nonfat milk with 0.1% Tween-20 for an hour on a shaker at room temperature and then probed with primary antibodies in 5% NFM in TBST overnight at 4°C and then incubated with secondary antibodies for 1 h at room temperature. The optical density of each band on the blot was quantified with ImageJ (NIH, Bethesda, MD), normalized to GAPDH and presented as fold change relative to the reference tissue.

### Statistics

Data are presented as means ± standard error of means. Comparison between H-I and control groups was made with *t-*test with appropriate corrections for multiple comparisons. Comparison between the age groups was made with one-way ANOVA. Repeated-measures ANOVA, followed by *post-hoc* correction for multiple comparisons procedure were used in rate dependent depression of H-reflex.

## Results

### Muscle Hypertonia in Newborns After Antenatal H-I Has Active and Passive Components

**Figure 1:**
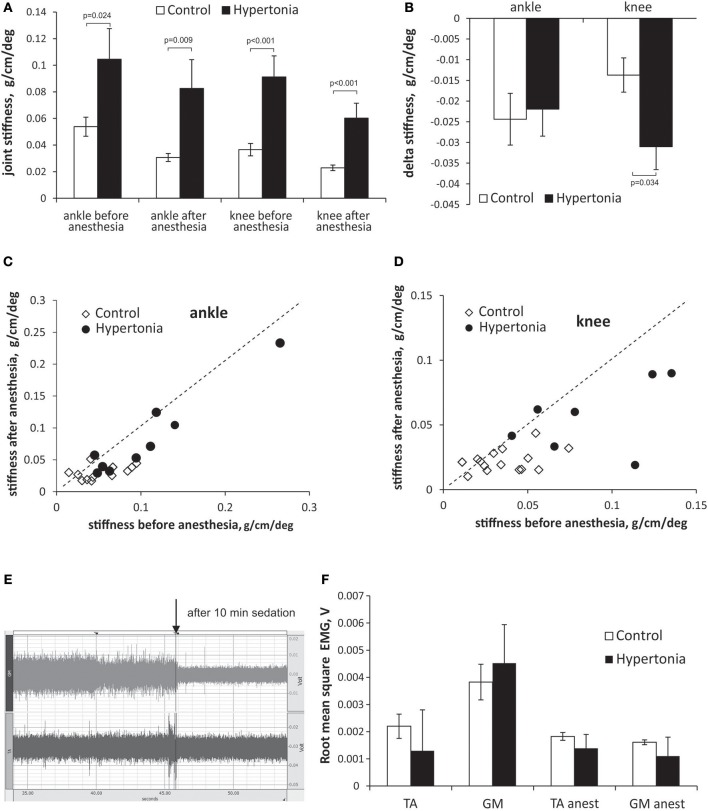
Evaluation of passive and active components in increased muscle tone in kits after antenatal H-I. Muscle tone was assessed using passive joint stretch. Absolute **(A)** and delta change **(B)** of joint stiffness is calculated before and after anesthesia in P1 kits with and without hypertonia. Individual animal joint stiffness before anesthesia in ankles **(C)** and knees **(D)** is plotted on horizontal axes, and after anesthesia on vertical axes. Dotted line represents the identity line. **(E)** Electromyogram in ankle extensors (m. gastrocnemius, GM) and flexors (m. tibialis anterior, TA) in a period without limb movement recorded using intramuscular barbed wire electrodes. EMG trace is shown in a hypertonic P1 rabbit before and after anesthesia (indicated by arrow) with Ketamine/Xylazine. **(F)** Root mean square EMG **(F)** decreased with anesthesia in TA and GM. No difference was founds between control and hypertonia groups before and after anesthesia.

The initial group assignment into hypertonic and non-hypertonic kits was based on manual scoring. We tested whether increased muscle tone, perceived by an investigator (25), corresponds to the laboratory measurement of passive joint resistance. Passive joint resistance in P1 kits was assessed by the stiffness component of torque -displacement response and was higher in hypertonic group than in non-hypertonic sham control group (Figure 1A). After Ketamine/Xylazine anesthetic administration, rendering kits to become flaccid and irresponsive to pinch, the joint stiffness decreased to < 0.05 g/cm/deg in non-hypertonic group. The joint stiffness in hypertonic group after anesthesia did not reach the stiffness level of non-hypertonic group, indicating presence of a passive component in increased muscle tone after antenatal H-I. The delta change of stiffness before and after the anesthesia was not different between the groups in ankles, but was larger in knees of hypertonic kits (Figure 1B), indicating presence of an active component to muscle hypertonia. Individual animal stiffness in knee and ankle joints before and after anesthetic administration is plotted on Figures 1C,D. There was some overlap in stiffness values between the hypertonic and control groups, likely due to incomplete relaxation in some kits and residual presence of active muscle resistance. The plot demonstrates that the animals with a larger stiffness in knees have a larger decrease of stiffness after the anesthetic.

To determine contribution of the baseline resting muscle activity (in the absence of active muscle contractions) to the increased joint resistance and postural deficits, EMG was recorded in ankle flexor and extensor muscles before and 10 min after anesthesia with Ketamine/Xylazine. The anesthesia caused a decrease in amplitude EMG in control and in hypertonic kits, as evident on EMG trace on Figure 1E. No difference between the control and hypertonic kits was found in mean RMS EMG before, as well as after the anesthesia (Figure 1F), indicating that neuronal component of muscle activity is present in hypertonic kits, but not increased compared to controls, and that the increase joint resistance in hypertonic kits cannot be explained by a persistent muscle activation or co-contraction of flexors and extensors.

### Muscle Architecture

**Figure 2:**
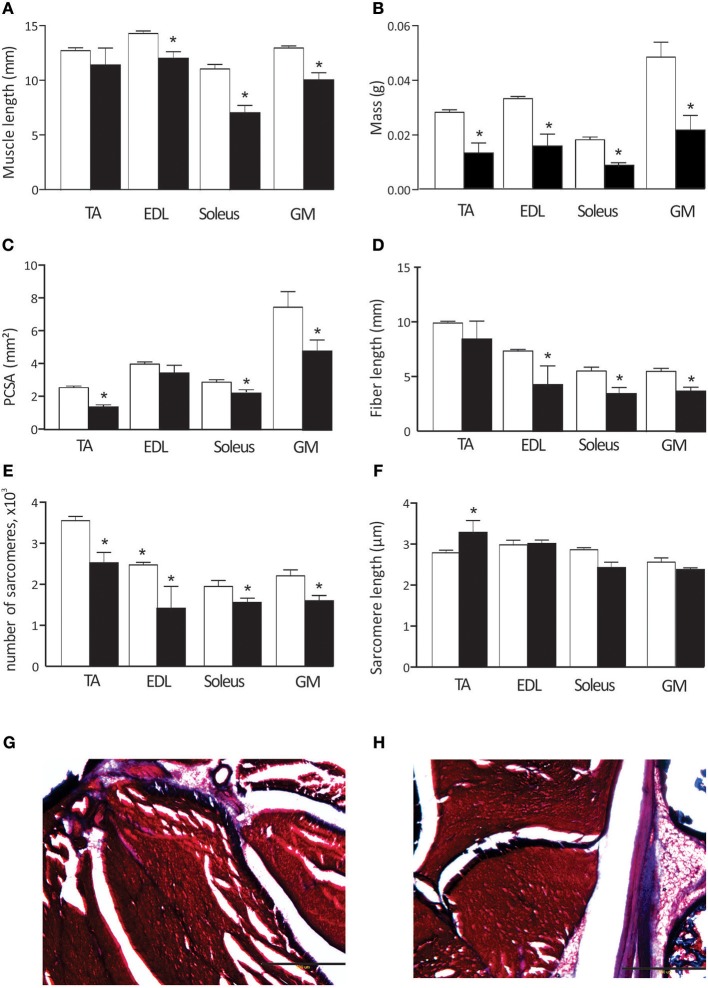
Muscle properties in control and hypertonic kits after antenatal H-I: Muscle length **(A)**, muscle wet mass **(B)**, physiological cross-sectional area (PCSA, **C**),muscle fiber length **(D)**, number of sarcomeres **(E)**, and sarcomere length **(F)**. Hind limbs were fixed in neutral position and dissected out. Masson trichrome staining on ankle muscles in control **(G)** and hypertonic **(H)** P1 kits, limb cross section: red–muscle fibers, blue–collagen and bone. TA, m. tibialis anterior; EDL, m. extensor digitorum longus; GM, m.gastrocnemius medius. Scale bar on **(G,H)** 500 microns. ^*^p < 0.05.

Hypertonic kits exhibit pronounced atrophy in limb muscles, evidenced by significant muscle shortening and decreased muscle mass (Figures 2A,B). Muscle fiber length and physiological cross-sectional area were also reduced in most muscles (Figures 2C,D), both agonists and antagonists. Serial sarcomere number was significantly lower in all studied muscles in hypertonic animals (Figure 2E). Sarcomere length was significantly longer in TA muscles (Figure 2F). Noticeably, hypertonic kits often present exaggerated permanent plantar flexion posture, consistent with the decreased serial sarcomere number and increased sarcomere length of the TA. We also stained limb muscle for connective tissue content to understand increased passive joint resistance in affected kits. Visual inspection of Masson trichrome stained muscles did not reveal apparent differences in extracellular matrix content (blue color in Figures 2G,H) between control and hypertonic kits.

### Maturational Changes in H-reflex in Normal Rabbit Development and After Antenatal H-I

**Figure 3:**
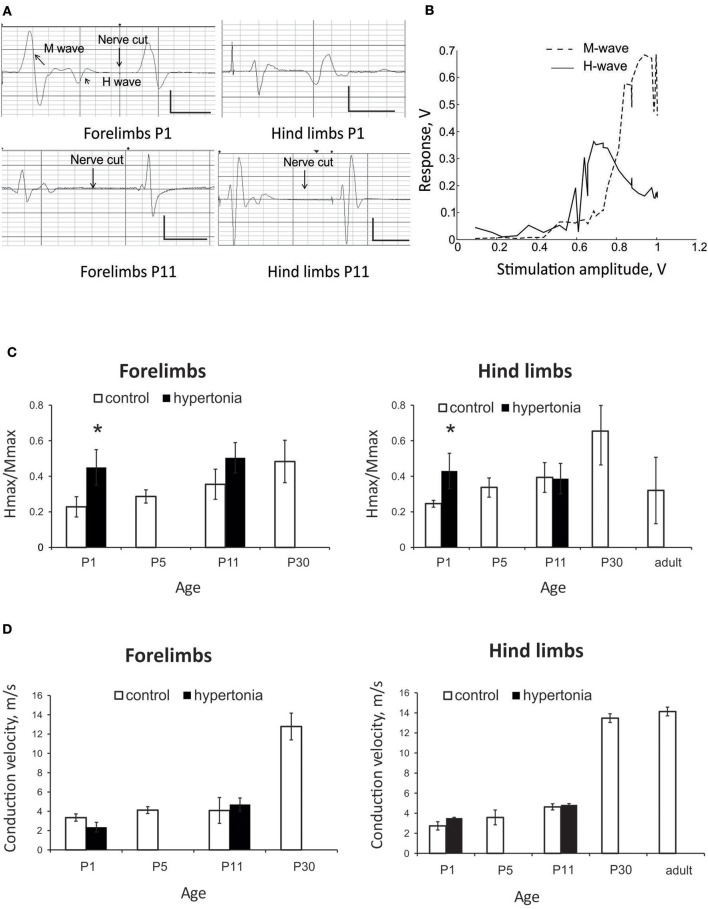
Assessment of monosynaptic H-reflex in development and after antenatal H-I injury. **(A)** Representative EMG recordings of nerve stimulation in forelimbs and hind limbs at P1 and P11 rabbit kits. Response consisted of M -wave with shorter latency and H-wave with longer latency. H-wave was abolished after nerve cutting (arrows) proximal to stimulation electrode. Calibration bars 0.5 V/10 ms. **(B)** Representative stimulation-response curve of H-reflex recorded on forelimbs of P5 control rabbit. Maximums of H- and M-waves were determined and Hmax/Mmax ratio. **(C)** Hmax/Mmax increased with age until P30 both in forelimbs and hind limbs in normal development. Hmax/Mmax was significantly increased with hypertonia at P1 but not at P11. **(D)** Conduction velocity increased with age, but was not altered in hypertonic animals. ^*^p < 0.05.

We were able to elicit H-reflex in 75% animals in forelimbs and 62% hind limbs at P1, and in almost all kits at later ages. Typical traces of the responses to the nerve stimulation in forelimbs and hind limbs are shown on Figure 3A. Nerve stimulation depolarizes sensory and motor fibers of mixed nerves bi-directionally, resulting in two wave types on EMG: the M-response (direct muscle activation) and the H-reflex (central loop pathway). Longer latency H-waves were abolished after the nerve cut proximal to stimulation site (arrows in Figure 3A), confirming spinal origin of the H-wave. While the M-wave amplitude increased progressively with the increments of stimulation current until it reached plateau, the H-wave reached maximal amplitude and then decreased with further increase of stimulation intensity (Figure 3B). Ratio Hmax/Mmax was increased with hypertonia in forelimbs (*p* = 0.032) and hind limbs (*p* = 0.041) at P1, but not at P11 (Figure 3C). Peripheral nerve conduction velocity gradually increased with development both in fore- and hind limbs with a rapid increase after P11 (Figure 3D). No difference in nerve conduction velocity was found between the control and hypertonia groups.

**Figure 4:**
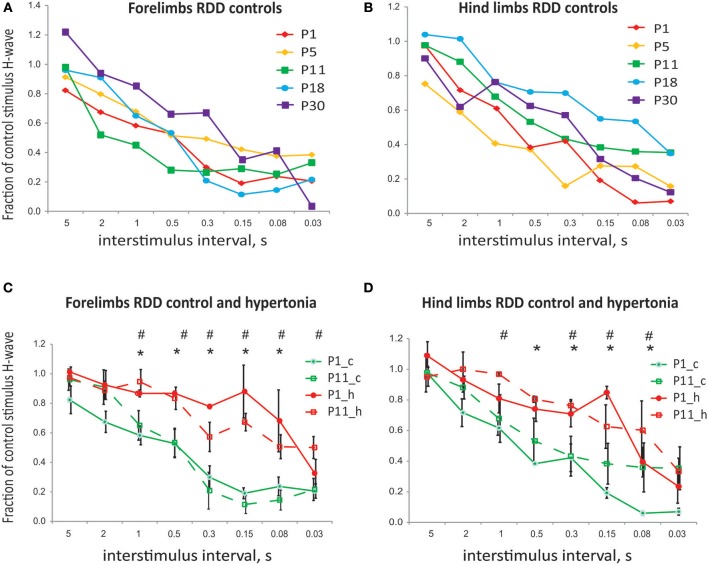
**(A)** Rate dependent depression of H-reflex in normal development and after antenatal H-I. No difference in rate dependent depression of H-reflex with development. **(B)** No difference in RDD between forelimbs and hind limbs for corresponding ages. **(C)** Reduction in RDD in hypertonic kits after H-I in forelimbs at P1 and P1. **(D)** Reduction in RDD in hypertonic kits after H-I in hind limbs at P1 and P1. ^*^p < 0.05 for P1, #*p* < 0.05 for P11.

In the test of rate dependent depression (RDD), the magnitude of the test pulse decreased with shorter intervals between the paired control and test stimulation pulses. There was no significant difference in RDD during normal postnatal development in tested ages between P1 and P30 in either forelimb or hind limbs (Figures 4A,B, error bars are not shown for clarity). There was also no significant difference in RDD between forelimbs and hind limbs in normal development at corresponding ages. Significant reduction of RDD was found in hypertonic kits after H-I, at P1 and P11, at inter-stimulus intervals 1 s and less, both in forelimbs (Figure 4C) and hind limbs (Figure 4D).

### Morphology of Motoneurons and Primary Afferent Fiber Density Are Not Changed After Antenatal H-I

**Figure 5:**
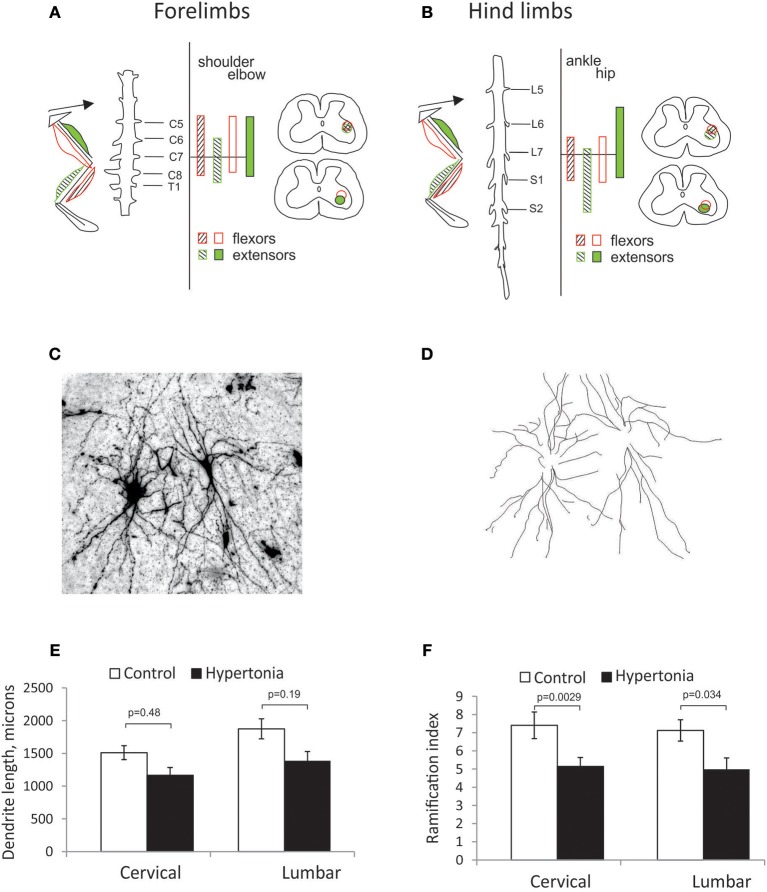
Somatotopic motoneuron mapping and dendritic tree sizes in motor neurons on Golgi staining. **(A,B)** Location of cholera toxin retrograde labeled neurons from distal and proximal muscle groups on spinal cord cross-sections in cervical **(A)** and lumbar expansions **(B)**. Location of motor neurons projecting to distal and proximal (wrist and elbow in forelimbs, ankle, and knee in hind limbs) muscles were indicated as hatched and filled circles, correspondingly, flexor, and extensor muscles—in empty and filled circles, correspondingly. Golgi stained motor neurons were photographed at 10x magnification as a stack and maximum intensity projection obtained **(C)**. Individual neurons were traced **(D)**. Sholl analysis was conducted to estimate length of dendritic tree **(E)** and ramification index **(F)**.

To identify location of spinal motor neurons projecting to the distal muscle groups where muscle hypertonia is typically observed in affected rabbit kits after antenatal H-I, labeling was conducted with retrograde neuronal tracer CTB injection in the corresponding muscle groups of forelimbs and hind limbs. Spatial distribution of labeled motoneurons relative to spinal segments is shown for forelimbs on Figure 5A and for hind limbs on Figure 5B. Location and spatial distribution of motoneurons was in general agreement with previously reported mapping in rabbit hind limbs (40). Motoneurons projecting to elbow and wrist muscles were reliably found between C6 and C8 segments and motoneurons projecting to hip and ankle—between L6/L7 and S1. Neurons of flexor muscles were found more dorso-medially relative to extensors, and neurons of proximal muscle—more medial relative to distal. While general somatotopy (41) was observed, there was a large overlap between the pools (Figures 5A,B). Therefore, a separate analysis of flexor/extensors and different joints was not conducted. Motoneurons in identified locations for forelimbs and hind limbs were traced on Golgi staining (Figures 5C,D) and the tracings were quantified on Sholl analysis. Hypertonic groups had smaller dendritic tree length [*F*_(1, 76)_ = 5.92, *p* = 0.017] and ramification index [*F*_(1, 76)_ = 20.4, *p* < 0.001], but *post-hoc* comparisons revealed significant differences between the groups for corresponding regions only for ramification index (Figures 5E,F).

**Figure 6:**
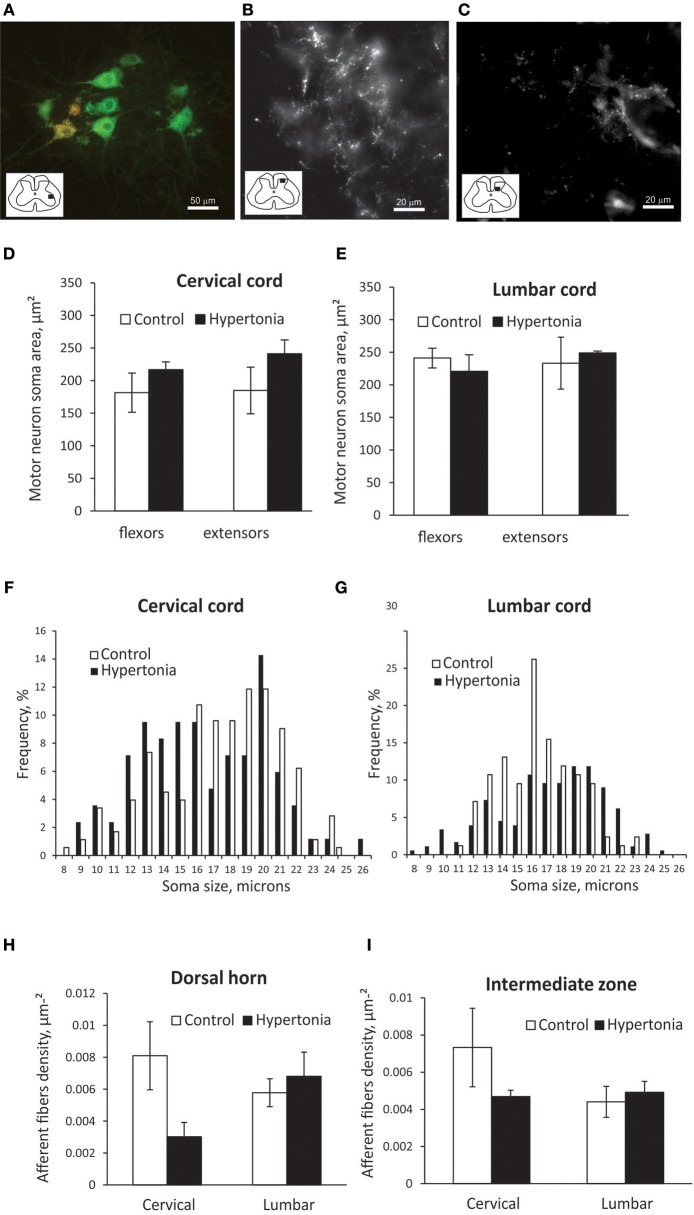
Labeling of motor neurons and primary afferents by intramuscular injection of CTB in newborn kits. Retrograde neuronal tracer cholera toxin B conjugated with different color fluorophores (orange and green) was injected in flexor and extensor muscle groups in wrist and ankle at P1 kits and cords were collected after 2 days. **(A)** Labeled motor neurons in ventral horn of cervical cord. Orange label is from wrist flexors and green label is from wrist extensor injection. Stereological estimation of motor neuron soma size is on **(D,E)** and distribution of sizes **(F,G)**. Primary afferents labeling in dorsal horn **(B)** and intermediate zone **(C)** and stereological estimation of fiber density **(H,I)**. Inserts in **(A–C)** show spinal cord diagrams indicating locations where high magnification picture were taken.

Typical labeling of motoneuron cell bodies and primary sensory afferents from distal muscles is shown on Figures 6A–C. Motoneurons soma size was significantly smaller in cervical cord than in lumbar cord in control P1 kits, 183.1 ± 22.8 vs. 237.1 ± 7.3 μm^2^, *p* = 0.026). Motoneurons, labeled by CTB injection in flexor muscle group were located more medially from neurons labeled by injection in extensor muscles, with significant spatial overlap between the motor pools (Figure 6A). No differences in soma size between control and hypertonic groups were found in motoneurons innervating distal flexor or extensor muscles, either in cervical or lumbar cord (Figures 6D,E). No apparent differences in distribution of soma sizes were found between control and hypertonic groups (Figures 6F,G).

Primary sensory afferent labeling from muscle injection of CTB was concentrated in two patches, in the dorsal horn substantia gelatinosa area and in the intermediate zone (Figures 6B,C). Primary afferent density was assessed separately in those areas. There was no significant difference in primary afferent fiber density between control and hypertonic kits (Figures 6H,I).

### Changes in Potassium Chloride Transporter KCC2 Are Not Related to Hypertonia After Antenatal H-I

**Figure 7:**
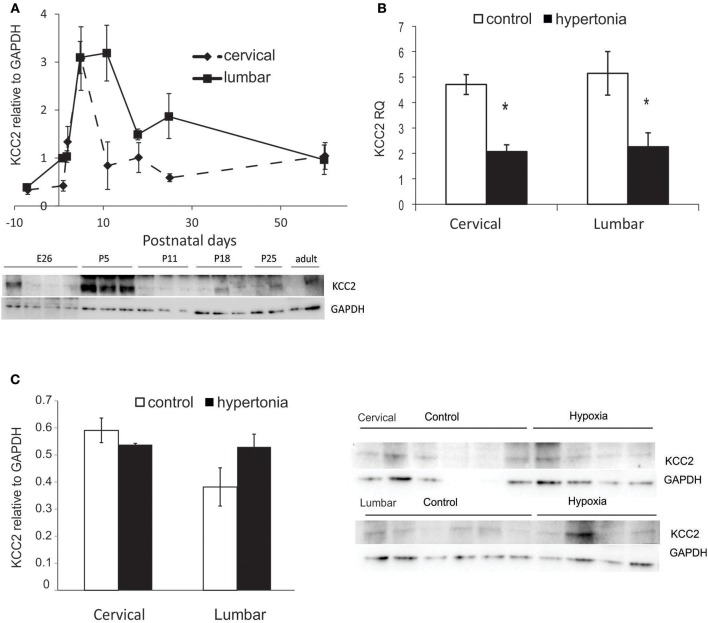
Expression of potassium chloride transporter KCC2 in normal development and after antenatal H-I injury. **(A)** KCC2 protein expression during development from fetal to adult ages in cervical and lumbar cords, quantified by optical density of KCC2 band relative to housekeeping protein GAPDH on Western blot. **(B)** Relative gene expression of KCC2 in control and hypertonic P1 rabbits on RT-PCR. **(C)** Western blot of KCC2 in control and hypertonic P1 rabbits. ^*^p < 0.05.

To examine if potassium chloride transporter KCC2 plays a role in motor deficits in affected rabbit kits after antenatal H-I, we first examined developmental changes in KCC2. Initially low KCC2 protein content in fetal naïve rabbit increased six-fold after birth, reached maximum around P5–P11 and then declined with maturation and reach plateau after P11–P18 (Figure 7A). Decrease of KCC2 in cervical cord was faster than in the lumbar, but the KCC2 content was not different between the cervical lumbar cord expansions in adult rabbits. Gene expression of KCC2 by RT-PCR was lower in hypertonic kits at P1 in cervical (*p* = 0.001) and lumbar (*p* = 0.018) (Figure 7B), but the protein content was not different between the control and hypertonic groups either in cervical nor in lumbar cord, measured on Western blot (Figure 7C).

### Increased Density of Astroglia Fibers and Activated Microglia in Spinal Gray Matter With Hypertonia

**Figure 8:**
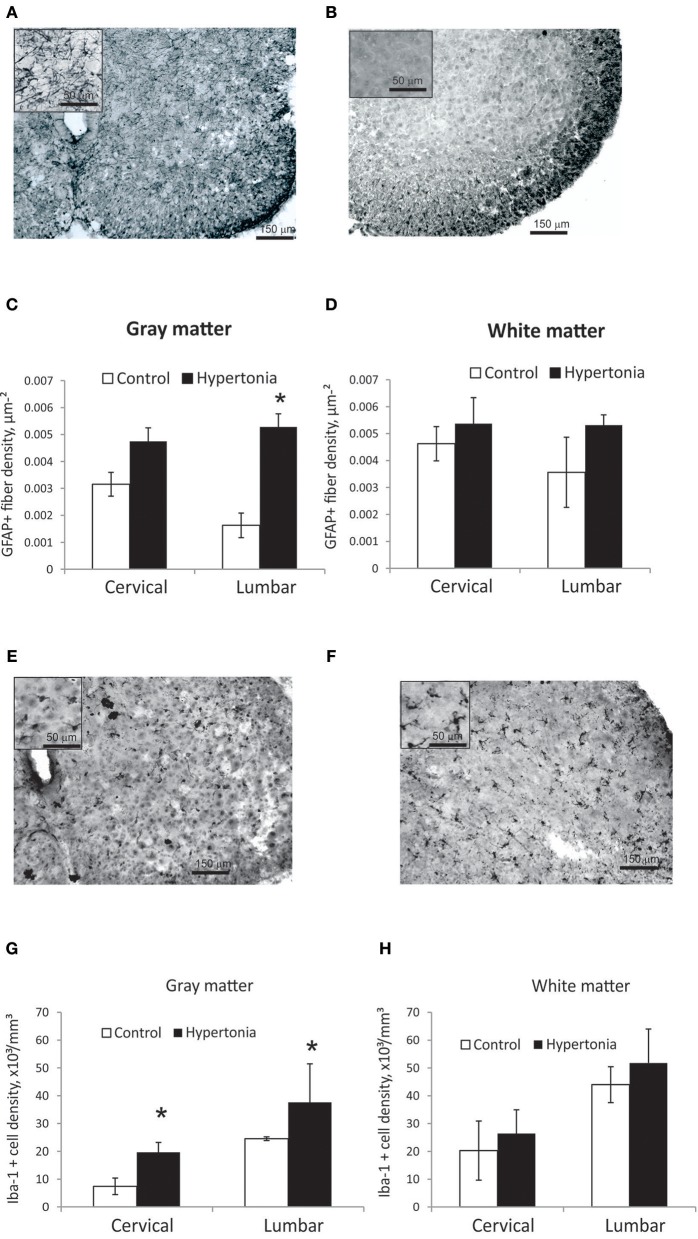
Astrogliosis and increased density of microglia in spinal cord gray matter after antenatal H-I injury. **(A,B)** Representative GFAP staining in cervical cord of control **(A)** and hypertonic **(B)** kits. Inserts on top left corners show glial fibers at higher magnification. Significant increase in glial fiber density in gray matter **(C)**, but not in white matter **(D)** found on stereological quantification of GFAP positive fiber length. **(E,F)** Representative Iba-1 staining in cervical cord of control **(E)** and hypertonic **(F)** kits. Inserts on top left show microglial cells at higher magnification. Note thickened cell bodies and shortened processed, indicative of activated microglia morphology, found in hypertonic as well as in control animals. Significant increase in number of Iba-1 positive cells in gray matter **(G)**, but not in white matter **(H)** on stereological quantification of Iba-1 positive cell density. ^*^p < 0.05.

Astroglia appeared on GFAP staining in spinal cord as predominately fibers with a few cell bodies (Figures 8A,B). GFAP positive fiber density was significantly higher in gray matter in hypertonic kits in lumbar cord (*p* = 0.014), but not different in white matter between the groups (Figures 8C,D). Astrogliosis was more pronounced in ventral horns of the hypertonic kits.

Iba-1 positive microglia in control as well as in hypertonic kits spinal cord had predominantly round and amoeboid shape with short thick processes (Figures 8E,F), typical for early postnatal period (42) and reminiscence of morphological appearance of activated microglia in older animals, in contrast to the quiescent, ramified microglia found in adults (43). Very few highly ramified Iba-1 positive cells were found in both groups; therefore only total number of Iba-1 positive cells was estimated on stereology. Number of Iba-1 positive cells was significantly higher in hypertonic group both in cervical and lumbar cord gray matter (Figures 8G,H) (p-val = 0.023).

## Discussion

In the current study we examined several mechanisms that could contribute to increased muscle tone in newborn rabbits after global antenatal H-I. The main findings can be summarized as follows: while evidence of decreased supraspinal inhibitory control and increased excitability in monosynaptic spinal reflex were found by decreased rate dependent depression and increased Hmax/Mmax in H-reflex, abnormal posture and increased joint resistance to stretch was explained primary by the biomechanical changes of muscles and joints.

### Hyperexcitability of Spinal Network

The monosynaptic Hoffmann reflex is considered a major probe for non-invasive study of sensorimotor integration in spinal cord in human and animals (44). The ratio of maximal amplitudes of H to M waves is commonly used as an index of peripheral reflex excitability (45). While excitability state of the α-motoneuron pool plays a significant role in determining the H-reflex magnitude (46, 47), it is also depends on multiple pre-and post-synaptic inhibitory mechanisms (31). Increased excitability of H-reflex monosynaptic pathway has been reported in children with cerebral palsy (33) with a positive correlation between Hmax/Mmax ratios and Modified Ashworth Scale. H-wave latencies were found to be shorter and Hmax/Mmax amplitude ratios were higher in children with spasticity compared to healthy controls. Using dependence of H-reflex on stimulation frequency, it is possible to infer about descending presynaptic inhibition (48). A marked decrease of RDD of the H-reflex was observed in children with spasticity and athetosis, but not with ataxia, studied in newborn children up through age 9 years, compared to typically developing children (32).

In our study we found significant changes in H-reflex properties after antenatal H-I. Increased Hmax/Mmax ratio and RDD reduction in hypertonic rabbit kits were similar to H-reflex changes observed in animals with ablation of corticospinal input in neonatal period using either surgical or genetic methods (20, 49) and to human patients with the spastic or dystonic types of CP (32, 33). RDD reduction in hypertonic kits suggests decreased supraspinal inhibitory control that might be mediated by the loss of descending motor projections after fetal H-I (27, 28). Increased Hmax/Mmax in newborn rabbits with hypertonia may also indicate that stronger afferent synaptic connections are retained in hypertonic kits due to injury or delayed arrival of corticospinal projections (10), but we did not find evidence for this based on quantification of sensory afferent density. Subsequent Hmax/Mmax decrease observed at later age in hypertonic kits may indicate continued degeneration of the spinal circuit.

In this study we characterized development of H-reflex in rabbits from early neonatal period to adulthood. While reports of H-reflex properties in hind limbs of rats are abundant, especially in spinal cord injury models, characterization of H-reflex is rare or limited in developmental settings, in forelimbs (50) and in rabbits (51). In our experience, H-reflex was easier to register in forelimbs in newborn at P1, suggesting faster development of forelimbs than hind limbs. While Hmax/Mmax increased with age, in agreement with previous reports in humans, no significant differences were found between forelimbs and hindlimbs in Hmax/Mmax and RDD in development. In typically developing children, RDD was less in infants aged 0 to 12 months compared to children aged 1–9 years, especially at inter-stimulus intervals ranging from 100 to 800 ms (32), and this was attributed to immature supraspinal inhibitory control.

### Skeletal Muscle Changes

To determine whether increased spinal neuronal excitability resulted in increased muscle activity, potentially explaining characteristic posture and increased joint resistance in affected rabbit kits, we quantified joint stiffness and EMG before and after anesthetic administration. Joint stiffness in the hypertonic group decreased after anesthetic administration by approximately the same amount as in control group in ankles and slightly more in knees. There was no change in baseline resting spinal output before and after anesthetic administration (as indicated by RMS EMG in resting muscle), a parameter frequently chosen because it reflects the level of motor unit activity (52). These observations demonstrate that increased joint resistance in the hypertonic kits is likely explained by changes in biomechanical properties of muscles and joints. In hypertonic kits we found a decrease in physiological cross—section area, muscle shortening, decreased serial sarcomere number, and an increased sarcomere length in some muscles. Importantly, these changes are observed in human CP patients (3, 53) and animals after neonatal deafferentation on spinal motoneurons (54). We did not find increased connective tissue, as observed in human spastic CP muscles (55, 56), but this may be explained by the age studied (P1 newborns) and may be develop in later ages as an adaptation of muscles to increased stretch.

In human adolescents with CP, both tissue stiffness, representing non-neuronal component, and reflex torque, representating neural contributor to joint stiffness were significantly increased in quantitative ankle testing and were proportional to a clinical measure of motor impairment (57). It is possible in the rabbit model, that increased spinal motoneuron and muscle activity may occur at earlier stages of injury *in utero*, followed by neuronal degeneration and not observed postnatally. Muscle shortening may occur secondary to hypertonia and lead to contracture formation that occurs with the growth of the long bones. The recent observation that the muscle stem cell population in decreased in children with CP is also consistent with this hypothesis (58, 59).

Observations in human CP presented evidence of slowed conduction velocity in children with spastic CP (60), implying that abnormalities in myelination may arise early in CP and persist into adulthood. In our study peripheral nerve conduction velocity increased with age in controls, but was not different to hypertonic kits at P1 and P11. Although we did not study peripheral conduction velocity till full maturation, unaltered nerve conduction velocity at P11, when the sciatic and median nerves are already largely myelinated in rabbits, indicate that the hypertonia in the rabbit model is due to injury in central nervous system and not peripheral.

### Morphological Changes That May Contribute to Intrinsic and Synaptic Properties of Motoneurons

High input resistance and low capacitance of immature motoneurons due to smaller size, less developed dendritic arborization and lower channel density, compared with adults, has been implicated in increased excitability at rest (61, 62). While in normal development, activity dependent increase of soma size and dendritic branching leads to a decrease in motoneuron excitability, perinatal injury may delay maturation of passive membrane properties. This may result in increases in cell input resistance and lower rheobase current, similar to changes occurring in motoneurons after spinal cord injury (63, 64), and contribute to the increased muscle tone at rest. We did not find evidence of altered soma size, length of dendrites and index of branching in motoneurons of newborn rabbits with hypertonia, suggesting that the changes in passive membrane properties in motoneurons may have limited contribution to the motor phenotype. We did not however examine channel density and number of synapses on motoneurons in our study.

Another possible explanation of increased excitability of spinal network after perinatal injury would be a disturbance of normally occurring developmental switch of GABA/glycine action from excitatory to inhibitory due to perinatal upregulation of chloride extruders, especially in potassium chloride transporter KCC2 (61). Loss or delay of KCC2 upregulation and deficiency to develop hyperpolarizing chloride potential occurs after injury to descending projections, as observed in neonatal cord transection (65) and after various kinds of neurotrauma (23). Downregulation in KCC2 expression have been implicated in origin of spinal hyper-excitability after spinal cord injury (66). Loss of KCC1 and KCC2 chloride transporters expression was found in brains of infants with white matter lesions (67). Hypertonic rabbit kits in our study did not have a loss of KCC2 protein contents in spinal cord at P1, but had lower KCC2 gene expression. The developmental profile, that we constructed, demonstrates that in rabbits KCC2 expression is low in fetuses but increases several fold during early postnatal period. Since motor deficits are already observed in newborn rabbits, we conclude it is unlikely that KCC2 disturbance contributes to the development of muscle hypertonia in the rabbit model of antenatal H-I injury, but may play a role in human CP where motor deficits develop usually postnatally.

A recent study on a rat model of fetal hypoperfusion (68) demonstrated persistent increase of motoneuron excitability based on decreased RDD of H-reflex, similar to data in our rabbit data. Neuronal hyper- reflexivity has been attributed to decreased expression of KCC2 in spinal cord of P8 rats after prenatal hypoperfusion. Apparent differences in the role of KCC2 in contribution to motor deficits between this model and the rabbit model may be related, apart from the species differences, to the timing of onset and the nature of motor deficits. In contrast to rabbits, motor deficits in the rat model were detected in later development, mild in nature and detected by gate abnormalities (69), and may be related to KCC2 action that occurs postnatally. Velocity dependent increase of joint resistance, i.e., spasticity, is briefly mentioned in this model (69) and in a model of neonatal anoxia with limb restriction (70) but remains to be demonstrated by objective measurements. In our previous publications we reported that the sensory and motor deficits persist at least to P11 (71) and P18 (28) in affected kits without improvement and apparent change in motor phenotype. We did not find indications of spasticity in qualitative (25) and in quantitative laboratory measurement of velocity dependent muscle tone at P1 (36).

Increased excitatory synaptic input from sensory afferents to motoneurons may contribute to increased muscle tone. Normal maturational retraction of sensory afferents in spinal cords occurs due to competition with descending motor projections (72) that can be disturbed after functional or structural injury to the descending projections (20, 73). We did not find histological evidence for this mechanism in our study since the density of primary afferents was not increased in hypertonic rabbit kits. This can be explained by the timing of tissue examination that was done in newborn rabbits at P1 and may precede the retraction of primary afferents, occurring postnatally and depending on activity in descending motor projections, as described in cats (16, 73).

Consistent finding among different animal models resulting in spinal hyperexcitability is gliosis in spinal cord, likely to represent glial reaction to neuroinflammation and neurodegeneration after central nervous system injury. Gliosis is described in model of H-I brain and spinal cord injury (34), including therabbit model of antenatal H-I (74), spinal cord injury (75), and corticospinal tract injury (18). Blocking astrocyte specific AMPA receptors alleviated symptoms of spasticity and rigidity after ischemic spinal cord injury (34). In the current study we report increased astro- and microglia in spinal gray matter of hypertonic kits. Multiple mechanisms have been suggested to explain how astroglia and activated microglia may affect spinal neuronal excitability, including release BDNF, TNFα, cytokines, and promoting reactive sprouting. It is unclear, however, whether gliosis is a primary cause of hypertonia in the rabbit H-I model or a secondary reaction to neuroinflammation, while also contributing to spinal hyperexcitability and motor deficits.

There are several important limitations that need to be addressed in this study. First, the number of animals per group was relatively low due to practical difficulties raising sick kits to older ages. The studied ages for affected animals was limited to P11 for H-reflex and to P1 for the rest of measurements because of already fully expressed and permanent motor deficits in newborns after E25 H-I injury (26). It is possible that we did not observe kits long enough to detect spasticity that commonly occur in humans in later development. Extending study to later postnatal period would allow examination of specific mechanisms relevant to human CP, such as changes in sensory afferents density or KCC2 expression.

Second, the studied groups were restricted to hypertonic animals and controls. There is a subpopulation of newborns in the rabbit model that exhibits decreased muscle tone, or flaccidity, that can be transitory and later either replaced by hypertonia or persist (36). Heterogeneous motor phenotype in newborns after global antenatal H-I injury in rabbits reflects complexity of human CP disease, where manifestation of motor deficit types depends on timing, severity and site of injury, and represent combination of brain injury (27, 28), direct spinal cord injury (74) and disrupted development. Due to compressed time scale of the rabbit development in comparison to humans, motor deficits in rabbits may develop relatively early, even in prenatal period. The types of motor deficits in rabbits are not completely equivalent to human CP, the most noticeable difference being lack of spasticity (25). Spasticity, which is attributed to postnatally developing alteration of intrinsic motoneuron excitability, may develop later in rabbits but can be obscured by developed contractures, muscle overstretching and atrophy.

It appears that the rabbit model of global antenatal H-I injury captures several salient features of human CP disease, such as pronounced and persistent motor deficits, including hypertonia and rigid posture, white and gray matter brain and spinal cord injury and abnormal muscle fiber properties. It is important that it models the injury that occurs in the antenatal period since majority of human CP, irrespective of type, is antepartum in origin (76). Antepartum H-I is thought to be one of the most important contributions in the etiology of spastic CP (77). The current study emphasizes the heterogeneous mechanisms contributing to motor deficits in CP, one of them being spinal hyperexcitability, which needs to be put in the specific developmental context of studied species.

## Author Contributions

SS, JL, LY, IG, RL, and AD participated in acquisition, analysis or interpretation of data for the work. AD conceived the work. AD, RL, and JL were drafting the work and revising it critically for important intellectual content.

### Conflict of Interest Statement

The authors declare that the research was conducted in the absence of any commercial or financial relationships that could be construed as a potential conflict of interest.
